# Obesity, white adipose tissue and cancer

**DOI:** 10.1111/febs.17312

**Published:** 2024-11-04

**Authors:** Estel Solsona‐Vilarrasa, Karen H. Vousden

**Affiliations:** ^1^ The Francis Crick Institute London UK

**Keywords:** cancer, obesity, white adipose tissue

## Abstract

White adipose tissue (WAT) is crucial for whole‐body energy homeostasis and plays an important role in metabolic and hormonal regulation. While healthy WAT undergoes controlled expansion and contraction to meet the body's requirements, dysfunctional WAT in conditions like obesity is characterized by excessive tissue expansion, alterations in lipid homeostasis, inflammation, hypoxia, and fibrosis. Obesity is strongly associated with an increased risk of numerous cancers, with obesity‐induced WAT dysfunction influencing cancer development through various mechanisms involving both systemic and local interactions between adipose tissue and tumors. Unhealthy obese WAT affects circulating levels of free fatty acids and factors like leptin, adiponectin, and insulin, altering systemic lipid metabolism and inducing inflammation that supports tumor growth. Similar mechanisms are observed locally in an adipose‐rich tumor microenvironment (TME), where WAT cells can also trigger extracellular matrix remodeling, thereby enhancing the TME's ability to promote tumor growth. Moreover, tumors reciprocally interact with WAT, creating a bidirectional communication that further enhances tumorigenesis. This review focuses on the complex interplay between obesity, WAT dysfunction, and primary tumor growth, highlighting potential targets for therapeutic intervention.

## Graphical abstract

White adipose tissue (WAT) is essential for energy balance and hormone regulation. In obesity, WAT becomes dysfunctional, leading to inflammation and other changes that promote different stages of carcinogenesis. This review explores how unhealthy WAT under obese conditions supports carcinoma growth through both systemic effects and modulation of the local tumor microenvironment. We discuss the bidirectional interaction between tumors and WAT, highlighting promising targets for cancer treatment. Figure created with Biorender.com.

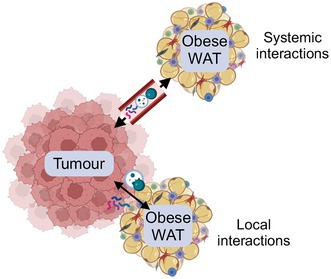

**Abbreviations**
AKT: protein kinase B
ASCs: adipose‐derived stromal/stem cells
AT: Adipose tissue
BAT: brown adipose tissue
CAAs: cancer‐associated adipocytes
CLS: crown‐like structures
CXCL: C‐X‐C Motif Chemokine Ligand
ER: estrogen receptor
EVs: extracellular vesicles
FAM3C: FAM3 metabolism‐regulating signaling molecule C
FAO: fatty acid oxidation
FFAs: free fatty acids
HCC: hepatocellular carcinoma
IGF‐1: insulin‐like growth factor‐1
IL: interleukin
JAK: Janus kinase
JNK: Jun N‐terminal kinase
LCFA: long‐chain fatty acids
MAPK: mitogen‐activated protein kinase
MASLD: metabolic dysfunction‐associated steatotic liver disease
MCP‐1: monocyte chemoattractant protein‐1
MMPs: matrix metalloproteinases
mTOR: mammalian target of rapamycin
NF‐κB: nuclear factor kappa B
NK cells: natural killer cells
PDAC: pancreatic ductal adenocarcinoma
PGE2: prostaglandin E2
PI3K: phosphoinositide 3‐kinase
SAA: serum amyloid A
SREBP: sterol regulatory element‐binding protein
STAT3: signal transducer and activator of transcription 3
SVF: stromal vascular fraction
sWAT: subcutaneous WAT
TAGs: triacylglycerols
TAMs: tumor‐associated macrophages
TGF‐β: transforming growth factor beta
TME: tumor microenvironment
TNF‐α: tumor necrosis factor‐alpha
VEGF: vascular endothelial growth factor
vWAT: visceral WAT
WAT: white adipose tissue

## White adipose tissue in health and disease

Adipose tissue (AT), otherwise known as body fat, is a central metabolic organ regulating whole‐body energy homeostasis. AT is broadly distributed across the body in several distinct depots, found mostly under the skin, packed around internal organs, and between muscles. Two main types of AT have been described: white adipose tissue (WAT) and brown adipose tissue (BAT). WAT, the focus of this review, is the most abundant, representing more than 95% of adipose mass [1, 2].

### Cellular, functional, and anatomical heterogeneity of WAT

**Fig. 1:**
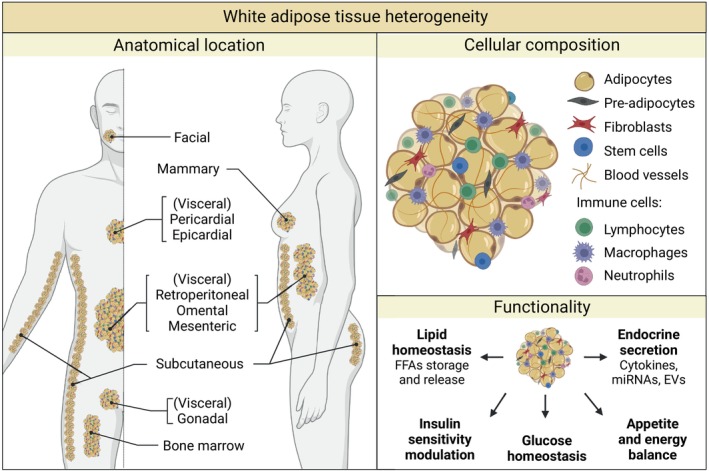
White adipose tissue (WAT) heterogeneity. WAT is a highly heterogeneous organ in terms of anatomical location, cellular composition and functionality. Anatomical location: Adipose tissue is organized into discrete depots throughout the body. Subcutaneous WAT is found beneath the skin, while visceral fat is located around internal organs. Major visceral depots in humans comprise epicardial/pericardial, retroperitoneal, omental, mesenteric and gonadal WAT. Other smaller WAT depots include the bone marrow and mammary fat. Cellular composition: Besides adipocytes, WAT is also composed of pre‐adipocytes, ASCs and a wide range of immune cells among others. Functionality: Apart from storing and releasing energy in the form of fat, WAT has an additional role as an endocrine organ, and it is also involved in insulin and glucose homeostasis as well as appetite and energy balance regulation. Created with BioRender.com.

WAT is diverse with respect to its cellular composition, functional characteristics, and anatomical location (Fig. 1) [3, 4]. About one third of the cells within WAT are fat cells known as adipocytes, which store energy in the form of triglycerides under conditions of energy surplus and mobilize it during fasting or periods of high energy demand. Additionally, adipocytes can produce and secrete bioactive molecules that affect nearby cells and other organs (Fig. 1). Besides adipocytes, WAT also contains other cell types such as preadipocytes, endothelial cells, fibroblasts, blood cells, macrophages, a rich collection of other innate and adaptive immune cells, and adipose‐derived stromal/stem cells (ASCs) with multipotent capacity. These non‐adipocyte cell types are known as the stromal vascular fraction (SVF) and can also produce active factors that act in a paracrine or endocrine manner on other cells and tissues [2, 5, 6]. Pre‐adipocytes originate from ASCs and have the potential to develop into mature adipocytes as part of a mechanism known as adipogenesis. This process occurs during the entire lifespan of an organism [7, 8].

Functions of WAT in energy balance, as an endocrine organ and in maintaining proper glucose homeostasis, are well established [9, 10, 11]. In the WAT, adipocytes store lipids via free fatty acids (FFAs) uptake or by converting excess glucose into acetyl‐CoA which is then converted into lipids via *de novo* lipogenesis, processes driven by the hormone insulin [1, 2, 12, 13]. When required, adipocytes can mobilize their lipid reservoirs through lipolysis, breaking down triacylglycerols (TAGs) into FFAs and glycerol, the first being used for oxidative phosphorylation in oxidative tissues and the second for gluconeogenesis in the liver. The catecholamines epinephrine and norepinephrine as well as other hormones such as leptin, cortisol, and glucagon stimulate lipolysis during fasting or exercise [14]. More recently identified, the endocrine activity of WAT reflects its ability to secrete a variety of molecules with both paracrine and endocrine actions. These contribute to many different physiological processes such as the regulation of nutritional intake, insulin sensitivity, and angiogenesis. These molecules include miRNAs, lipids, cytokines, peptide hormones, and extracellular vesicles (EVs) such as exosomes. WAT‐released factors include tumor necrosis factor‐alpha (TNF‐α), interleukin (IL)‐6, monocyte chemoattractant protein‐1 (MCP‐1), and serum amyloid A (SAA), as well as leptin and adiponectin, two signaling molecules almost exclusively produced by adipocytes (therefore also named adipokines) [15, 16].

Anatomically, WAT is categorized into subcutaneous (sWAT) and visceral (vWAT) types. sWAT, which comprises 80% or more of total fat mass in humans, is located beneath the skin where it stores lipids and acts as an insulator to prevent heat loss and as a barrier against infection. vWAT, comprising 5–20% of body fat, surrounds and protects internal organs as well as releases FFAs into circulation. Additional small deposits of fat are broadly distributed across the body associated with organs such as muscle, breast, and bone marrow, serving mechanical and signaling roles [1, 2, 17, 18]. Notably, fat depot function and structure differ between species, making it essential to consider these differences when translating animal studies to humans [19].

Importantly, WAT heterogeneity goes beyond the differences described above. For example, the metabolic behavior and secretory patterns of WAT are largely dependent on its anatomical site, and each fat depot is unique in terms of cellular composition. Furthermore, there is heterogeneity among adipocytes, even within the same WAT depot [20, 21]. Such heterogeneity is a challenge not only for adipose tissue research but also for the study of the role of WAT in disease.

### Adaptation and maladaptation in WAT

**Fig. 2:**
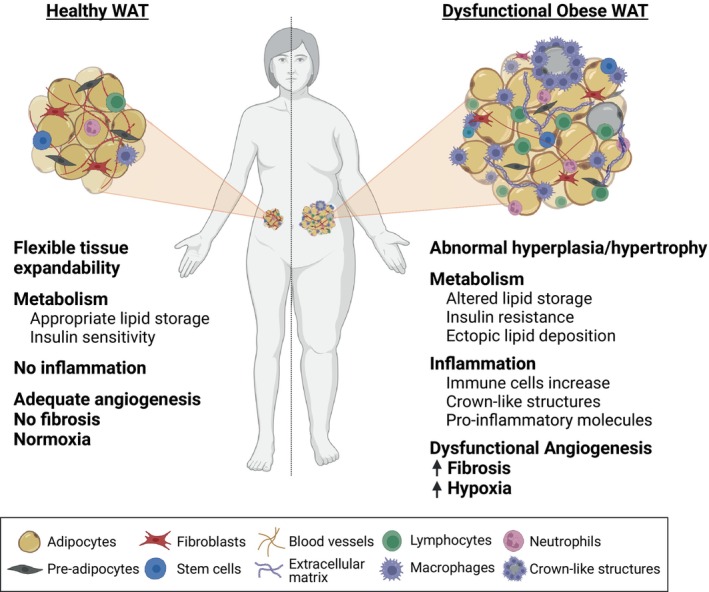
Hallmarks of healthy and dysfunctional obese WAT. Healthy WAT is identified by its flexible expandability through controlled hyperplasia and/or hypertrophy, adequate lipid storage and responsiveness to insulin. It exhibits minimal inflammation, high vascularization, and lacks fibrosis and hypoxia. By contrast, in the context of obesity, compromised WAT displays abnormal expandability and lipid storage, insulin resistance, and increased release of lipids that accumulate in other organs. Additionally, it releases numerous pro‐inflammatory molecules leading to the recruitment of pro‐inflammatory immune cells and the formation of adipose crown‐like structures. Besides inflammation, dysfunctional obese WAT is further characterized by reduced angiogenesis, increased hypoxia, and fibrosis attributed to the excessive production of extracellular matrix components. Created with BioRender.com.

WAT needs to rapidly shift from one metabolic state to another to meet the requirements of the body under conditions such as feeding, fasting, cold exposure or exercise—switching from storing to releasing FAs. This constant WAT expansion and contraction over an individual's lifetime is tightly regulated by neuronal and hormonal signals. Healthy WAT expansion is characterized by a controlled increase of adipocyte number (hyperplasia) and/or size (hypertrophy), low inflammation, and high vascularization required for proper WAT oxygenation, as well as controlled nutrient and hormone transport to and from the tissue [22, 23] (Fig. 2).

Despite the extraordinary adaptability of WAT, there are limitations to this plasticity that lead to metabolically dysfunctional WAT and metabolic disorders. Unhealthy WAT usually presents with excessive tissue expansion through hyperplasia and/or hypertrophy, insufficient angiogenesis leading to hypoxia, fibrosis from excessive deposition of extracellular matrix components, and chronic inflammation through the uncontrolled production of pro‐inflammatory cytokines and immune cells infiltration (Fig. 2). These alterations in WAT can also drive insulin resistance and lipid accumulation in other non‐adipose organs. In sum, these changes have been linked to the development of metabolic diseases such as obesity, type 2 diabetes, cardiovascular diseases, hepatic steatosis, and cancer [22, 23, 24]. Interestingly, the pathological effects of increased or decreased adipose tissue are similar. Lipodystrophy, which is characterized by partial or generalized absence of body fat, also results in insulin resistance, ectopic fat depots, and metabolic syndrome [25, 26].

Many different factors contribute to WAT maladaptation and the consequent metabolic syndrome, including genetic and lifestyle factors such as diet and exercise, the location of the affected WAT, the quality of the fat (for instance whether inflammation or hypoxia are present), and the manner of tissue expansion. While hypertrophic growth is linked to high inflammation and hypoxia, hyperplastic growth seems to be more metabolically favorable [22, 23, 24]. Dysfunctional WAT is commonly found in obesity, a complex health issue caused by an imbalance between energy intake and expenditure. The connection between obesity, WAT dysfunction, and their impact on the progression of cancer is a topic of increasingly active research investigation.

## Association between WAT, obesity, and cancer

### Obesity as a cancer risk factor

Cancer ranks among the primary global causes of mortality, and projections suggest an escalating mortality rate in the years ahead. The etiology of cancer occurrence and progression is complex and multifactorial. While much progress has been made in understanding the tumor intrinsic changes that occur during malignant progression, more recently the importance of tumor‐host interactions has also become evident, identifying cancer as a systemic disease [27].

Epidemiologic data strongly associate obesity with the development of numerous different cancer types [28, 29, 30] with some studies suggesting that obesity and smoking carry similar risks for cancer development [31, 32]. Carcinomas, including pancreatic and breast cancers, make up the majority of cancer types linked to obesity and have been most closely studied. Additionally, there is a clear association between obesity and multiple myeloma, a type of cancer that originates in plasma cells in the bone marrow. This link has recently been reviewed by experts in the field [33]. There is also evidence linking obesity to other hematological cancers, such as leukemias [34], and certain sarcomas [35, 36], although further research is needed to fully understand these connections. Therefore, in this review, we focus on the impact of obesity on epithelial tumors.

Considering the alarming increase in obesity rates worldwide and the high mortality caused by cancer, it is imperative to understand and fully establish the connection between obesity and cancer. It is clear, however, that this is a complex question, and although there is a strong correlation between excess adiposity and increased risk of cancer, there are also exceptions to it. Obese individuals that are otherwise metabolically healthy and show low levels of inflammation seem to be at low risk for presenting specific obesity‐associated cancers. Conversely, the risk is higher in lean individuals with more inflammation and metabolic derangements [37, 38]. These metabolically obese but normal‐weight patients are clinically identified by presenting multiple metabolic derangements such as hypertension, dyslipidemia or elevated plasma glucose levels, that are known to be risk factors for cardiovascular disease and diabetes [39, 40, 41]. Overall, this suggests that the metabolic and inflammatory status of the adipose tissue may be more important than the overall amount in influencing the risk for certain cancer types.

### Impact of obesity on cancer

The excessive adipose tissue expansion through hypertrophy and hyperplasia in obese patients is accompanied by other characteristics of dysfunctional WAT including altered lipid storage, insulin resistance, ectopic lipid accumulation, local hypoxia, and increased reactive oxygen species [42, 43, 44, 45, 46]. In such conditions, adipocytes are more likely to die, worsening tissue inflammation due to the recruitment of pro‐inflammatory immune cells, the formation of “crown‐like structures” (CLS)—consisting of macrophages surrounding dead adipocytes—and increased release of pro‐inflammatory molecules. As described in the next section, these alterations in the WAT under conditions of obesity can support primary tumor growth.

Cancer growth in obese patients can also involve changes in the intestinal microbiome and the direct effects of obesogenic diets *per se*. Abnormal perturbations in the gut microbiota and dysfunctional gut barrier play a key role in the obesity‐cancer link. Amino acids, lipids, and other metabolites as well as toxins such as lipopolysaccharides are among the gut microbiota‐derived factors that are altered in obesity [47]. This increase in nutrient availability and toxic metabolites that can be carcinogenic leads to metabolic derangements that promote cancer progression [48]. The unrestricted transfer of microbial metabolites to the liver due to gut dysbiosis has been linked to hepatic inflammation, fibrosis, and progression to hepatocellular carcinoma (HCC) [49]. The type of lipid available in the diet can also be a key determinant of tumor progression [50], with evidence that different obesogenic diets can have diverse impacts on cancer development, despite causing the same degree of obesity. Dietary influences on tumor progression occur, in part, by altering the access to and utilization of nutrients by cancer cells [51]. In addition, nutrients from the diet can also impact immune cells—in some cases leading to an immunosuppressive environment that favors tumor growth. A high‐fat diet, commonly used to study obesity, induces alterations in lipid metabolism that are associated with dysfunction in various immune cells, including CD8^+^ T cells [52, 53], natural killer (NK) cells [54, 55], and dendritic cells [56], hampering their anti‐tumor ability.

While we focus here on primary tumor growth, obesity has been reported to support all stages of carcinogenesis, from cancer initiation to metastasis [57]. The elevations of plasma FFAs derived from dysfunctional WAT result in an accumulation of lipids in other organs such as the liver and the pancreas. The build‐up of fat in the liver causes metabolic dysfunction‐associated steatotic liver disease (MASLD), a condition associated with fibrosis and cirrhosis that can eventually progress to HCC [58]. The pancreas can also exhibit fat deposition (fatty pancreas), which has been suggested to contribute to the development of pancreatic cancer [59]. Furthermore, obesity plays a role in cancer metastasis. Through altered levels of cytokines, insulin, and extracellular vesicles—among other factors—the obese state promotes cancer cell de‐differentiation and facilitates the release of cancer cells from the primary tumor. It also supplies blood vessels for cancer cells spread and primes distant niches to support invasion. Several recent reviews cover this important aspect of the link of obesity with metastasis [57, 60, 61]. Obesity also leads to difficulties in cancer diagnosis and treatment [62, 63, 64, 65]. Obese patients are more likely to develop surgical complications and an increased death rate following cancer surgery and may receive inadequate chemotherapy and radiotherapy dosages [62, 63]. While this holds true in many cases, further complexity is introduced by the “obesity paradox”, where some obese patients respond better to treatments such as immunotherapies compared to their normal‐weight counterparts [66]. This counterintuitive finding has been observed not only in cancer but also in other chronic disorders such as cardiovascular disease [67]. Untangling these connections is confounded by the multiple factors that are influenced by obesity, including the misclassification bias caused by using BMI as an obesity measure [68]. The relationship between obesity and cancer initiation, progression, diagnosis, and treatment therefore remains a subject of ongoing investigation.

## WAT dysfunction, obesity, and cancer progression

**Fig. 3:**
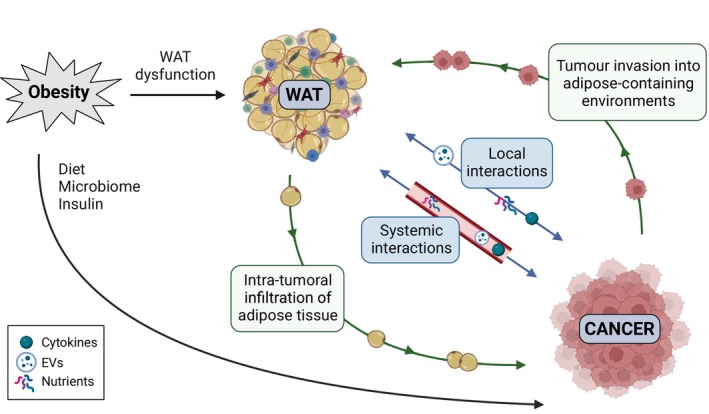
Impact of obesity on WAT‐cancer interactions. Some cancers are locally affected by WAT depots as they are embedded or in close contact with them. WAT also mediates endocrine effects through the bloodstream, impacting not only those cancers directly connected to fat depots but also others that lack such proximity. Certain tumors can also interact with cells from adipose tissue that infiltrate the tumor environment. Furthermore, specific cancer types can spread to the omental fat and adipose‐containing sites such as the bone marrow. Obesity can fuel cancer progression not only through various independent mechanisms such as diet and the microbiome but also by directly altering WAT function, thus creating a more favorable environment for tumor growth. Created with BioRender.com.

Numerous interactions have been observed between WAT and tumors, which are amplified in the context of obesity (Fig. 3). WAT can act locally and systemically to drive and support cancer progression. Direct WAT‐tumor interactions are observed in many cancer types such as breast, prostate, kidney, and melanoma that grow embedded or in immediate contact with WAT. Besides acting locally, WAT also mediates endocrine effects, allowing it to have an impact on additional cancer types that have no direct contact with a major fat depot, such as endometrial, liver, and pancreatic cancers [69, 70, 71]. While the influence of proximal WAT may be more profound than that of distal WAT, the obesity‐associated risk of developing cancer does not seem to correlate with whether the tumor is directly in contact with WAT. For example, the increased risk of breast cancers (embedded in WAT) is similar to pancreas cancer (where the influence of WAT is more likely to be distal), while endometrial cancer (no direct contact with WAT) shows the largest increased risk with obesity [29]. However, it should be noted that some cells from the WAT have the ability to traffic and invade organs that are not normally embedded in WAT. For example, an increased adipocyte content has been observed in human pancreatic tumors, with studies reporting an association between adipocytes infiltration into the pancreas and the development of pancreatic ductal adenocarcinoma (PDAC) [72]. Similarly, ASCs have been reported to traffic and invade tumor sites, as observed in prostate cancer [73].

In addition to the ability of adipose tissue to influence cancer, it is evident that tumors can also influence WAT, thereby creating a bidirectional communication between these two tissues. Tumors clearly reprogram WAT that is within or near the tumor stroma [74], but they can also remodel and benefit from more distant WAT depots. An example of this is cancer cachexia, a complex metabolic syndrome mainly characterized by tumor‐induced systemic inflammation, and loss of muscle, body weight, and AT [75, 76]. Cachexia is highly debilitating for the patient and can limit therapy and survival, but there is also evidence that the breakdown of normal tissue can enhance tumor growth [77]. Release of the exosomal miR‐204‐5p by breast cancer cells has been recently reported to induce leptin signaling, promoting WAT lipolysis and browning in cancer‐associated cachexia [78]. There are numerous further examples of tumor types that drive the release of lipids from adipocytes to satisfy their metabolic requirements, a subject of intensive research in breast cancer [79]. Furthermore, some types of cancer spread to adipose‐containing environments. For example, breast and prostate cancers can metastasize to the bone marrow, where they interact with the AT found in this tissue [80, 81]. Other solid tumors that show local invasion into the omental vWAT are the intra‐abdominally metastasizing ovarian, gastric, colorectal, and pancreatic cancers [82].

Taken together, the crosstalk between WAT and cancer creates a more tumor‐supporting environment, which is further enhanced by obesity.

### Systemic effects

**Fig. 4:**
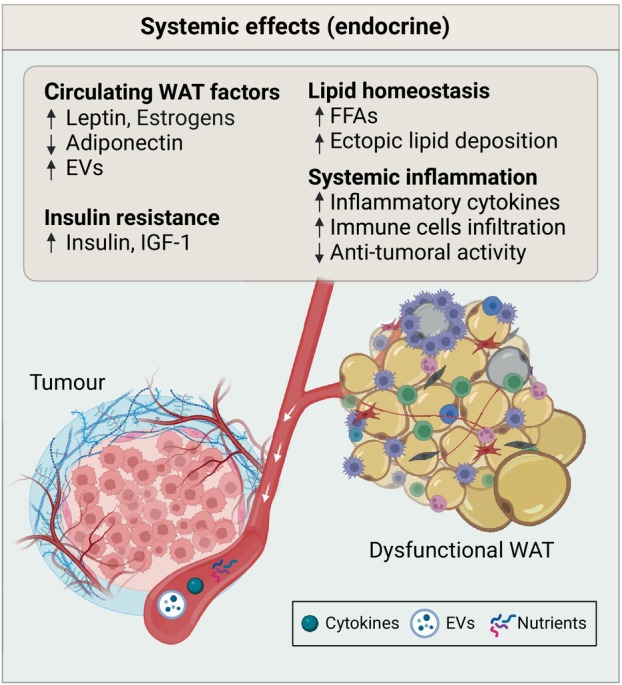
Main systemic effects of dysfunctional WAT on tumor progression. Dysfunctional WAT systemically impacts cancer progression mainly by inducing changes in the circulating levels of WAT‐derived factors and insulin resistance, altering the systemic lipid metabolism, and triggering an inflammatory state. Created with BioRender.com.

As mentioned above, even cancers that do not directly interact with WAT can be influenced by WAT and obesity. Dysfunctional WAT, present under obese conditions, leads to systemic changes that can impact cancer progression by inducing modifications in the circulating levels of WAT‐derived factors and insulin signaling, altering systemic lipid metabolism and triggering an inflammatory state (Fig. 4) [83, 84]. Other mechanisms linking WAT dysfunction with cancer development include a systemic increase of oxidative stress, a subject that has been recently reviewed by Jovanović and colleagues [85].

#### Effects of obesity on circulating WAT‐derived factors and insulin signaling

WAT maladaptation leads to altered systemic levels of different cytokines, growth factors, and hormones that are known to act as signals that promote tumor cells growth. It has been proposed that dysfunctional WAT contributes to the pathophysiology of cancer by raising systemic levels of leptin, steroid hormones, insulin, and insulin‐like growth factor‐1 (IGF‐1) while lowering the concentration of adiponectin [69, 71].

Leptin is an anorexigenic (or appetite suppressing) hormone that is produced in response to elevated lipid storage in the WAT, signaling to the brain to inhibit hunger and promote an increase in energy consumption [86]. Leptin interacts with its receptor ObR and activates pro‐survival pathways in cancer cells via effects on signaling through the Janus kinase (JAK)/signal transducer and activator of transcription 3 (STAT3), extracellular signal‐regulated kinase (ERK), protein kinase B (AKT) and Jun N‐terminal kinase (JNK) pathways [87]. High levels of circulating leptin are found in obese patients and are thus favorable for the growth of tumors, which in certain cases—such as HCC—can upregulate the expression of ObR to boost the oncogenic effects of leptin signaling [88]. Adiponectin physiologically acts as an anti‐inflammatory hormone and increases sensitivity to insulin in tissues such as the liver and the skeletal muscle [89]. Besides this function, there is significant evidence supporting an antineoplastic role of this adipokine in different types of cancer including breast and gastric cancers [90, 91]. In contrast to leptin, adiponectin levels are reduced in obese individuals, a response that is beneficial for tumor growth [92, 93]. Enhanced leptin and reduced adiponectin levels have been reported to trigger hepatic steatosis, activating inflammation and fibrosis that can lead to liver cancer [58].

The plasma concentration of specific steroid hormones such as estrogens is also increased upon WAT dysfunction and may favor the progression of certain cancer types. Estrogens have been reported to promote tumor development through several mechanisms, including the stimulation of cell proliferation, the inhibition of apoptosis and the induction of angiogenesis [94, 95, 96]. The therapeutic targeting of aromatase, the enzyme responsible for a key step in the biosynthesis of estrogens, has been demonstrated to be among the most effective ways to treat and prevent the recurrence of estrogen‐dependent cancers such as estrogen receptor (ER) positive breast cancers [97]. The link between estrogens and obesity‐associated colorectal cancer is less clear, as it has been reported that estrogens may reduce the risk of colorectal cancer [98].

Another critical consequence of unhealthy WAT in obesity is insulin resistance and elevated insulin levels in the circulation. Together with insulin, WAT malfunction is also characterized by increased levels of IGF‐1. Both insulin and IGF‐1 can induce the activation of specific oncogenic pathways including the phosphoinositide 3‐kinase (PI3K)/AKT/mammalian target of rapamycin (mTOR) and the mitogen‐activated protein kinase (MAPK) pathways [99, 100], which have multiple effects on cancer progression such as promoting obesity‐associated hepatic tumorigenesis [58]. The activation of PI3K/AKT signaling pathway in obesity has also been linked to colorectal and breast cancer [101, 102]. High circulating levels of insulin can also inhibit the hepatic synthesis of the steroid‐hormone‐binding globulin, causing further increases in the levels of free estradiol and androgens [69]. Several other WAT‐derived factors associated with the progression of breast and colon cancers, such as resistin, adipsin, and lipocalin‐2, remain to be fully understood [103, 104, 105, 106].

In addition to the classical adipokines, dysfunctional WAT can also modify the production and secretion of EVs into the bloodstream. By altering the quantity and composition of adipocyte‐derived EVs, WAT can transfer factors and nutrients to tumors that promote their aggressiveness and proliferation. Interestingly, other cells in the AT such as macrophages and ASCs also release EVs, mediating systemic WAT‐cancer interactions via different mechanisms [107].

#### Effects of obesity on systemic lipid metabolism

Dysfunctional adipose tissue shows diminished capacity to properly store lipids, leading to an elevation in circulating FFAs and an aberrant storage of lipids in other organs, alterations that have been demonstrated to play crucial roles in the formation and progression of diverse obesity‐associated carcinomas [71, 108]. The circulating FFAs can be taken up by cancer cells and are mainly used as a source of energy through fatty acid oxidation (FAO), although they can also be used for membrane synthesis and the generation of lipid‐derived signaling molecules that promote proliferation [69, 108]. Beyond FFAs, proteins that include the enzymes necessary for FAO can also be transported from WAT to distant tumors via EVs, thereby instigating metabolic remodeling in cancer cells and ultimately enhancing tumor aggressiveness [107, 109].

#### Effects of obesity on systemic inflammation and immune system response

During weight gain, adipocytes accumulate lipids, undergo hypertrophy, and eventually die, leading to the rupture of cell membranes and the release of cellular contents into the surrounding microenvironment. These released signals, including lipids, cytokines, reactive oxygen species, and nucleic acids among others, serve as major triggers for immune cell infiltration into the adipose tissue [70]. Macrophages accumulate in the dysfunctional WAT, with 10% of macrophages estimated to be present in the WAT of lean mice, compared to 50% in severely obese mice [110]. In the WAT, macrophages directly associate with dead or dying adipocytes, encircling them to form CLS [111]. Paracrine signaling between adipocytes and macrophages leads to increased secretion of pro‐inflammatory mediators, thereby exacerbating the inflammatory status in adipose tissue. Besides macrophages, other immune cells such as CD8^+^ T cells also infiltrate the WAT, contributing to increased WAT inflammation [17, 112].

The release of pro‐inflammatory factors from chronically inflamed WAT affects not only the adipose tissue itself but also has a systemic impact on other organs such as the liver, pancreas and brain, as well as distant tumors. IL‐6 is a cytokine involved in various biological activities such as hematopoiesis and immune regulation [113]. IL‐6 production by adipocytes is increased under obesity [114] and functions to support cell survival, proliferation, and angiogenesis. Elevated circulating levels of IL‐6 correlate with disease aggressiveness and poor prognosis across various cancer types [115]. TNF‐α, another pro‐inflammatory cytokine secreted by adipocytes and SVF cells, has the potential to drive tumor progression and is increased in the plasma of obese individuals [116]. HCC, for example, is associated with hepatic inflammation and activation of the STAT3 factor, conditions that are triggered by increased levels of IL‐6 and TNF‐α [117]. Activation of nuclear factor kappa B (NF‐κB) by increased levels of TNF‐α in obesity is also involved in colorectal carcinogenesis [118]. The production of IL‐1, a regulatory cytokine that promotes survival, proliferation, and angiogenesis, is also increased in obesity and associated with proliferation of cancers such as PDAC and breast carcinoma [119, 120].

The lipotoxic obese environment also leads to the impairment of systemic immunosurveillance against tumors. For example, obese people have fewer circulating NK cells compared to their lean counterparts. These NK cells suffer metabolic reprogramming, exhibiting aberrant activity and reduced cytotoxicity toward cancer cells [55, 121].

### Local effects

In addition to the systemic effects of obesity, cancers that arise in colocation with WAT may derive additional benefits that support tumor development. One critical determinant of oncogenic progression is the ability of cancer cells to acquire pro‐tumorigenic signals and nutrients from their immediate environment. These signaling and energetic needs are satisfied through several mechanisms, one of which is the alteration of the TME. Complex interactions occur between cancer and non‐cancer cells that surround the tumor, such as fibroblasts, immune cells and WAT [68, 122].

**Fig. 5:**
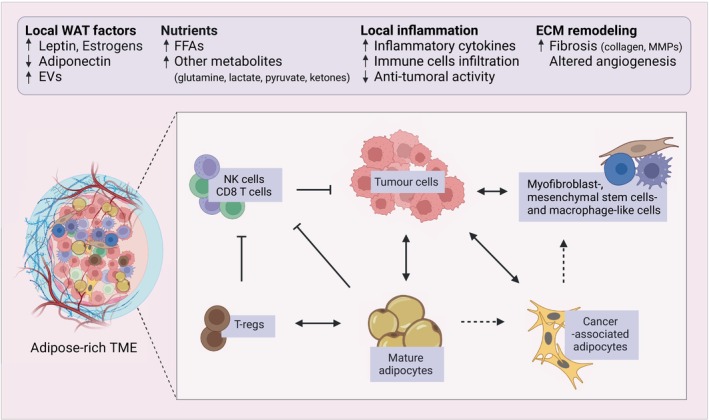
Major local effects of dysfunctional WAT on tumor progression. The local effects of unhealthy WAT involve abnormal release of adipokines and nutrients and local inflammation. In addition, aberrant WAT triggers extracellular matrix remodeling, generating a favorable microenvironment for tumor progression. In the adipose‐rich TME, there is also extensive crosstalk between cancer cells and different adipose‐derived cell types such as adipocytes and adipose‐derived stromal/stem cells (ASCs). Apart from mature adipocytes feeding the tumor, cancer cells can also induce the transformation of adipose cells into cancer‐associated adipocytes and later into myofibroblast‐, mesenchymal stem cell‐ and macrophage‐like cells to further satisfy their oncogenic needs. Cytokines and adipokines made by the adipocytes also act on immune cells present in the TME. Mature adipocytes can enhance T‐regs function and inhibit CD8^+^ T and NK cells activity, overall leading to decreased anti‐tumoral activity. Created with BioRender.com.

Within the TME, adipocytes can support tumor progression *per se*, but they are also locally conditioned by the tumor to further benefit tumor growth (Fig. 5). Tumor cells release a variety of soluble factors such as catecholamines, pro‐inflammatory cytokines, adrenomedullin, plasminogen activator inhibitor 1, WNT3a, and exosomal microRNAs, that result in the transformation of neighboring mature adipocytes into cancer‐associated adipocytes (CAAs). CAAs are characterized by undergoing delipidation and a decrease in size, downregulation of adipocyte differentiation markers, and alterations in the secretion of different factors, feeding cancer cells with numerous molecules that favor cancer survival [74, 123, 124, 125, 126, 127]. Prolonged CAAs stimulation involves additional morphological changes, causing them to undergo trans‐differentiation into various cell types, such as myofibroblast‐, mesenchymal stem cells‐ and macrophage‐like cells, all of which can further enhance cancer cell proliferation [128, 129, 130]. Such adipocyte plasticity has been reported not only in cancer research but also in other fields such as tissue regeneration and repair [131]. CAAs (which have been observed both *in vitro* and *in vivo*) were initially described in breast and ovarian cancers [123, 132], but have more recently been detected in other cancers such as pancreatic [133], lung [134], prostate [135], colorectal [136], and HCC [137, 138]. Other cells from the adipose tissue SVF found in the TME can also be altered by tumors to support cancer survival. ASCs have been reported to be recruited by cancer cells to the tumor site [139], where they exert local effects by supporting extracellular matrix remodeling, enhanced vascular endothelial growth factor (VEGF) production and angiogenesis [140]. C‐X‐C Motif Chemokine Ligand (CXCL)‐1 and ‐12 are among the chemokines implicated in the ASCs migration in prostate carcinoma [73, 141]. More recently, ASCs have also been reported to undergo fibroblastic differentiation in the TME in some carcinomas such as breast and PDAC [140, 142, 143]. It has also been shown that, in addition to inducing de‐differentiation of mature adipocytes into CAAs, tumors can also impair preadipocyte differentiation. Breast cancer cells have been recently reported to reprogram preadipocytes toward tumor‐protective immunomodulatory cells, a transformation that is significantly potentiated upon loss of p53 in cancer cells [144]. Similarly, cells from triple‐negative breast cancer secrete zinc‐alpha‐2‐glycoprotein, a modulator that inhibits adipogenesis and instead promotes the trans‐differentiation of adipocyte stem cells into cancer‐associated fibroblasts (CAFs) that support tumorigenesis [145].

While the pathways described above are not specific to obesity, the effects of CAAs seem to be more profound in the setting of obesity. Tumor‐educated obese adipocytes release higher levels of signaling factors and lipids, offering valuable insights into the association between specific cancer types and their heightened clinical severity in obese conditions. In common with the systemic effects of obesity, the local effects of an aberrant WAT on cancer progression involve modifications of WAT‐derived factors, nutrients, and inflammatory mediators. Furthermore, dysfunctional WAT also triggers extracellular matrix remodeling, making the TME more favorable for tumor growth [71, 146, 147].

#### Changes in local nutrients and WAT‐derived factors

As seen with systemic changes, aberrant cells from the WAT can provide local signaling factors and nutrients to cancer cells (Fig. 5). Adipocytes and ASCs act as the local sources of leptin and adiponectin, with a high leptin‐to‐adiponectin ratio being reported within breast cancer TME [148, 149, 150]. Interestingly, ASCs isolated from obese individuals express higher amounts of leptin relative to the same cell type isolated from lean counterparts and the increase in leptin under obesity enhances the proliferation and metastasis of breast cancer cells [151]. WAT‐derived estrogens and EVs are also altered in the TME, where they play a pro‐tumorigenic role. Aromatase and estrogen expression in adipocytes is induced by prostaglandin E2 (PGE2) made by breast cancer cells and CAFs [152]. Tumor‐infiltrated macrophages can also release PGE2 upon stimulation by nutrient excess under obese conditions [153]. Induction of aromatase expression in CAAs is triggered by type I interferons, which are produced in the TME by tumor‐infiltrating immune cells, as well as cancer cells [154]. FAM3 metabolism‐regulating signaling molecule C (FAM3C), a factor overexpressed in breast and colon cancers that regulates various pro‐tumorigenic proteins such as RAS, STAT3 and the transforming growth factor beta (TGF‐β) [130], has recently been reported to be released by CAAs in the TME, promoting the survival of the breast cancer cells [155].

Not surprisingly, locally produced adipocyte‐derived lipids also play a crucial role in supporting cancers. CAAs undergo delipidation and transfer FFAs to neighboring cancer cells to induce metabolic reprogramming, growth and invasion in many different cancer types. Because of the increased lipid accumulation in adipocytes in the context of obesity, the impacts of CAAs are enhanced in obese individuals [70, 124]. Adipocytes from obese mice have been reported to release FFAs to breast cancer and melanoma cells, which increase FAO and lipid accumulation, and ultimately promote higher rates of cell proliferation and migration compared to the influence of adipocytes from lean mice [156]. While short and medium‐chain FFAs can passively diffuse across the plasma membrane, the transport of long‐chain fatty acids (LCFA) is facilitated by a protein‐regulated system. CD36, also known as fatty acid translocase, plays a role in WAT lipolysis by triggering the mobilization of LCFA from adipocytes and therefore increasing the availability of FFAs for cancer cells in the TME [157].

In addition to lipids, CAAs also trigger other catabolic processes by liberating macronutrients such as glutamine, lactic acid, pyruvate, and ketones [74, 138]. Glutamine secreted by CAAs plays a key role in pancreatic cancer by favoring tumor cell growth in nutrient‐poor conditions [158]. Adipocyte‐derived β‐hydroxybutyrate can promote the growth of breast cancer cells that express the monocarboxylate transporter 2 [159], although a more recent study has shown that, when induced by a ketogenic diet, this ketone functions to inhibit colorectal cancer [160]. The reason for these different responses is not yet clear. Adipocytes can also reprogram metabolism in proximal cancer cells via mechanisms other than providing nutrients. For example, CAAs can redirect cancer cells' glucose metabolism toward glycerol‐3‐phosphate generation, a process that is regulated by adipocyte‐induced HIF‐1α protein, and that aids ovarian cancer metastasis [161]. Adipose tissue‐derived preadipocytes have also been reported to locally support cancer cells. A recent study has shown that the release of creatine and creatinine by preadipocytes triggers mitochondrial Ca^2+^ uptake and growth of esophageal and breast tumor cells respectively [162]. Each of these activities of CAAs is likely to be exacerbated in obesity.

#### Changes in local inflammation and immune response

The chronic inflammation that is observed systemically is also seen in the TME under obese conditions. Adipocytes themselves, together with immune cells such as macrophages, regulate the inflammatory status of the adipose‐rich TME by releasing inflammatory factors such as IL‐1, IL‐6 and TNF‐α, all of which benefit tumor progression [127, 163, 164].

Obese adipocytes also shape the tumor immune microenvironment by affecting the effector functions of immune cells (Fig. 5). Under physiological conditions, immune cells that exhibit anti‐tumor responses, such as CD8^+^ T cells and NK cells, rely on glycolysis for their activity, while immune‐regulatory cells, like T‐regs, use FAO to suppress anti‐tumor immunity. However, in the low‐glucose and lipid‐rich TME of obese individuals, immune cell metabolism and function are affected, resulting in increased immunosuppression and ultimately fuelling tumor progression [165, 166, 167]. Glucose restriction and increased FAO in CD8^+^ T cells under obese conditions lead to the inhibition of their anti‐cancer responses, as observed in breast and pancreatic cancers [168, 169]. NK cell anti‐tumor activity is also impaired due to increased lipid uptake and FAO [170]. Macrophages in a high lipid environment exhibit increased FFA metabolism, leading to polarization toward a more tumor‐promoting phenotype [171, 172]. Recent research in pancreatic, renal, and breast cancer models has also shown that obesity‐related cytokines induce PD‐1 expression in tumor‐associated macrophages (TAMs), inhibiting their anti‐tumor activity. Interestingly, anti‐PD‐1 immune checkpoint blockade therapy can counteract this effect by reactivating the phagocytic function of TAMs, strengthening their capacity to trigger an anti‐tumor response and increasing the overall effectiveness of the treatment [173, 174]. For T‐regs, increased CD36‐regulated lipid uptake together with upregulation of the sterol regulatory element‐binding protein (SREBP) for lipid synthesis enhances their suppressive functions to promote melanoma and colon cancer growth [175, 176]. In addition to FFAs, increased release of cytokines such as IL‐6 and leptin by adipocytes in the TME can also lead to tumor immune escape, as observed in prostate cancer cells that are resistant to the cytotoxic action of NK cells [163, 177].

#### Changes in TME extracellular matrix

Finally, obesity is also associated with structural remodeling of the TME (Fig. 5). Tumors in close contact with dysfunctional WAT benefit from such tissue reorganization as changes in the TME matrix composition contribute to their survival and progression [70, 178]. Aberrant adipocytes are characterized by excessive deposition of extracellular matrix components such as collagen VI in the TME [179, 180, 181] along with enhanced release of matrix metalloproteinases (MMPs) [182, 183], changes that have been shown to promote tumor progression in numerous models. Other factors, such as VEGF, are also produced by adipocytes in response to high insulin levels and modulate angiogenesis in the TME [184]. Furthermore, adipokines such as leptin can induce the secretion of various MMPs by cancer cells as well as the angiogenic differentiation of endothelial cells, thereby indirectly promoting neovascularization and tumor progression [185, 186]. Obesity also promotes crosstalk between adipocytes, tumor‐associated neutrophils and pancreatic stellate cells to promote matrix remodeling and impaired vascular perfusion, resulting in tumor progression and ineffective chemotherapy delivery in PDAC [187]. In HCC, adipocytes have been reported to trigger endothelial cell tube formation and vascularization benefiting tumor growth [137]. In addition to adipocytes, ASCs have the capability to differentiate into cells resembling cancer‐associated fibroblasts, thereby generating extracellular matrix components that support PDAC and breast cancer growth [188, 189, 190].

## Future perspectives

Despite significant progress in our understanding of the cancer/adipocyte crosstalk and the impact of obesity on these interactions, much remains to be learnt. Here we have focused on the role of obesity and WAT in primary tumor growth. However, it is important to note that obesity also significantly impacts other critical processes, such as metastasis and tumor response to chemotherapy. Although most research indicates that obesity tends to be detrimental to cancer, the relationship between obesity and cancer is complex, as highlighted by the obesity paradox described above and the concept that metabolic fitness may be more important in determining disease than obesity *per se*. More specifically, normal adipocytes have been described to limit metastasis in some breast cancers [191] and activate the anti‐tumor response in PDAC TME [192], reflecting the suggestion that the quality of WAT – whether healthy or diseased – may have a greater impact than the quantity of WAT in driving cancer. It is also possible that CAAs show heterogeneity that mirrors that seen in mature adipocytes [193], with an accompanying variation in the effect on cancer progression. Recent scRNAseq studies have also revealed heterogeneity in other WAT populations such as ASCs [194], further complicating the field. A better understanding of the specific adipose tissue‐derived factors that promote or inhibit cancer growth, such as resistin, adipsin, and lipocalin‐2, may also help to resolve these issues.

At this point, our understanding of the adipocyte/cancer interactions indicates that targeting adipocytes may be an effective therapeutic option for many patients, with the possibility of specific opportunities in certain patient populations. For example, a recent study has shown that upregulation of miR‐1304‐3p, which stimulates CAAs to release lipids, is upregulated in African American patients [195], suggesting a mechanism underpinning the disproportionately high rate of breast cancer progression in this population. Therapeutic approaches would depend on the development of effective strategies to target CAAs and disrupt their pro‐tumorigenic functions, while minimizing potential adverse effects on normal adipose tissue and metabolic homeostasis. In light of the recent success of immunotherapy, a deeper understanding of how adipocytes shape the immune landscape of the tumor microenvironment will also lead to new treatment opportunities.

Finally, there are technical challenges with respect to the isolation and culture of adipocytes, with concern that the analysis of cells differentiated from pre‐adipocytes *in vitro* may not fully recapitulate the function of mature adipocytes *in vivo*. Progress with improving the survival of adipocytes in culture and the development of co‐culture models and organoids to recapitulate the complex adipocyte‐tumor cell interactions seen *in vivo* will greatly enhance our ability to carry out mechanistic studies and drug screening in a more physiologically relevant context.

While future studies are needed to further elucidate the complex role of unhealthy obese WAT on cancer progression, the rapid rate of progress in this area provides some optimism that new targets for effective interventions will soon be revealed.

## Conflict of interest

KHV is on the board of directors and shareholder of Bristol Myers Squibb and on the science advisory board (with stock options) of PMV Pharma, RAZE Therapeutics, Volastra Pharmaceuticals and Kovina Therapeutics. She is on the SAB of Ludwig Cancer and a co‐founder and consultant of Faeth Therapeutics. She has been in receipt of research funding from Astex Pharmaceuticals and AstraZeneca and contributed to CRUK Cancer Research Technology filing of patent application WO/2017/144877. The other authors declare no conflicts of interest.

## Author contributions

ES‐V and KHV wrote the review.
